# Evaluation of electroacupuncture as a non-pharmacological therapy for astrocytic structural aberrations and behavioral deficits in a post-ischemic depression model in mice

**DOI:** 10.3389/fnbeh.2023.1239024

**Published:** 2023-08-28

**Authors:** Jingwen Wang, Xin Deng, Jin Jiang, Zhengyu Yao, Yaxin Ju, Yong Luo

**Affiliations:** ^1^Department of Neurology, The First Affiliated Hospital of Chongqing Medical University, Chongqing, China; ^2^Laboratory Research Center, The First Affiliated Hospital of Chongqing Medical University, Chongqing, China; ^3^Department of Hepatobiliary Surgery, The First Affiliated Hospital of Chongqing Medical University, Chongqing, China

**Keywords:** post-ischemic depression, stress, astrocyte, electroacupuncture, branch morphology, depressive-like behavior, BCCAO, bilateral common carotid artery occlusion

## Abstract

**Background:**

Ascending clinical evidence supports that electroacupuncture (EA) is effective in treating post-ischemic depression (PID), but little is known about how it works at the cellular level. Astrocytes are exquisitely sensitive to their extracellular environment, and under stressful conditions, they may experience aberrant structural remodeling that can potentially cause neuroplastic disturbances and contribute to subsequent changes in mood or behavior.

**Objectives:**

This study aimed to investigate the effect of EA on behavioral deficits associated with PID in mice and verify the hypothesis that astrocytic morphology may be involved in this impact.

**Methods:**

We established a PID animal model induced by transient bilateral common carotid artery occlusion (BCCAO, 20 min) and chronic restraint stress (CRS, 21 days). EA treatment (GV20 + ST36) was performed for 3 weeks, from Monday to Friday each week. Depressive- and anxiety-like behaviors and sociability were evaluated using SPT, FST, EPM, and SIT. Immunohistochemistry combined with Sholl and cell morphological analysis was utilized to assess the process morphology of GFAP+ astrocytes in mood-related regions. The potential relationship between morphological changes in astrocytes and behavioral output was detected by correlation analysis.

**Results:**

Behavioral assays demonstrated that EA treatment induced an overall reduction in behavioral deficits, as measured by the behavioral Z-score. Sholl and morphological analyses revealed that EA prevented the decline in cell complexity of astrocytes in the prefrontal cortex (PFC) and the CA1 region of the hippocampus, where astrocytes displayed evident deramification and atrophy of the branches. Eventually, the correlation analysis showed there was a relationship between behavioral emotionality and morphological changes.

**Conclusion:**

Our findings imply that EA prevents both behavioral deficits and structural abnormalities in astrocytes in the PID model. The strong correlation between behavioral Z-scores and the observed morphological changes confirms the notion that the weakening of astrocytic processes may play a crucial role in depressive symptoms, and astrocytes could be a potential target of EA in the treatment of PID.

## 1. Introduction

In clinical settings, ischemic stroke not only leads to sensorimotor disability during the acute period but also brings about long-term psychological complications. Among the various types of mood disturbances, post-ischemic depression (PID) is the major neuropsychiatric consequence that affects one-third to two-thirds of stroke survivors and is associated with relapsing neurovascular events, poor functional rehabilitation, and an elevated increase in mortality (Singer et al., 2021; Li et al., 2022). To date, the pathogenesis and pathophysiological mechanisms underlying PID remain poorly understood. However, two main causes, namely, the direct biological damage from brain ischemia and external environmental stimuli, such as chronic stress from lifestyle changes, may synergistically contribute to brain-cellular maladaptation and the subsequent behavioral changes (Farajdokht et al., 2022; Frank et al., 2022).

Astrocytes are the most abundant glial cell type in the central nervous system (CNS). They not only nourish neurons metabolically and regulate neuronal activity by buffering ions and neurotransmitters but also are involved in synapse formation (Diaz-Castro et al., 2021). Recently, a growing body of evidence from post-mortem and preclinical studies reveals that astrocytic abnormalities are closely linked to stress-related psychiatric disorders (Zhou X. et al., 2019). Interestingly, compared with the controversial changes in cell density, subtle morphological alterations that occur in astrocytic branching processes have been frequently reported to respond to stress and are supposed to mediate depressive-like behaviors (Zhou X. et al., 2019; Codeluppi et al., 2021). In reality, astrocytes playing their role in synaptic function highly rely on the processes that protrude outward and have varying lengths. For example, the typical “tripartite synapse,” in which astrocytic processes wrap tightly around pre- and post-synaptic neuronal elements, provides a platform for astrocytes to release paracrine substances known as “gliotransmitters,” such as adenosine triphosphate (ATP), glutamate, and D-serine, to bidirectionally interact with neurons (Singer et al., 2021). Therefore, from a morphological perspective, the changes in the process are more likely to be a “visual mirror” reflecting astrocytes’ state and structural plasticity which can affect neuron functions, particularly in pathological conditions. Of note, although studies on major depressive disorder (MDD) may provide insights into the mechanisms and therapeutic strategies for PID as they may share similarities in some aspects (Singer et al., 2021; Pagonabarraga et al., 2023), unfortunately, the structural pathology of astrocytes during PID has been poorly studied previously, and it remains unclear whether astrocytic processes are altered in this illness.

Electroacupuncture (EA) is an essential complementary and alternative therapy that combines traditional Chinese acupuncture with modern electrotherapy. With a deeper understanding, researchers are gradually recognizing the anatomical foundation and therapeutic mechanisms for EA treatment (Torres-Rosas et al., 2014; Liu et al., 2021; Yao et al., 2023). Previous studies conducted by us and others have demonstrated that EA stimuli can effectively alleviate cerebral ischemic injury and promote spontaneous recovery through various mechanisms, including suppressing neuroinflammation and oxidative stress, promoting cerebral-blood flow and neurogenesis, and generating cerebral ischemic tolerance (Jung et al., 2016; Jiang et al., 2017; Xing et al., 2018; Yao et al., 2023). Besides, almost all antidepressants [selective serotonin reuptake inhibitors (SSRIs), serotonin and noradrenaline reuptake inhibitors, and monoamine oxidase inhibitors] are associated with increased risks of adverse outcomes, including mortality, suicide, hemorrhagic complications, bone fractures, and seizures (Li et al., 2022), and patients may exhibit treatment-refractory response and poor adherence to antidepressant drugs. Increasing attention has been paid to the application of EA for alleviating depressive symptoms after stroke, in light of their few side effects and lower cost (Kang et al., 2021; Hu et al., 2023). However, despite the thriving evidence proving the remarkable therapeutic efficacy of EA for PID in clinical practice and randomized controlled trials, there are considerably fewer basic experiments regarding EA’s effects at the cellular level, particularly on neural cell types besides neurons, and this, to some extent, may limit our understanding of the mechanism of EA.

In this study, we attempted to find evidence supporting the hypothesis that EA protecting PID may be related to its impact on modulating astrocyte structure. To this end, we established a PID animal model by bilateral common carotid artery occlusion (BCCAO) and chronic restraint stress (CRS) to mimic the depressive state that often occurs after global cerebral ischemia (GCI) in humans (Farajdokht et al., 2022). Afterward, we examined behavioral assays including sucrose preference test (SPT), forced swim test (FST), social interaction test (SIT), and elevated plus maze test (EPMT) by performing Z-score analyses, not only to analyze anhedonia, despair, social withdrawal, and anxiety which are the core symptoms of PID (Verma et al., 2014; Koga et al., 2020; Cardinal et al., 2021) but also because Z-score analyses can provide a reliable means to normalize, combine, and compare behavioral results that have distinct units (Guilloux et al., 2011). Additionally, Sholl analysis and main morphological characteristics, including the number of branch processes, total process length, and the number of endpoints (Tynan et al., 2013; Lin et al., 2023), were utilized to assess the process morphology of GFAP+ astrocytes in mood-related regions. Finally, we examined the potential relationship between morphological changes in astrocytes and behavioral consequences. Altogether, our current findings may help to fill a gap in understanding astrocyte morphology in PID and EA therapy.

## 2. Materials and methods

### 2.1. Animals

Male C57BL/6J mice (aged 7–8 weeks) and retired male CD-1 mouse (aged 16 weeks) were obtained from the Experimental Animal Centre of Chongqing Medical University (Chongqing, China). All animals weighed 25–30 g and were housed in cages under standard conditions (a 23 ± 2°C ambient temperature, a humidity of 60 ± 5%, and a 12 h light/dark cycle). All experimental procedures were conducted in accordance with international ethical guidelines and were approved by the Ethics Committee of Chongqing Medical University. All efforts were made to minimize animals’ suffering.

### 2.2. Experimental design and procedure

**FIGURE 1:**
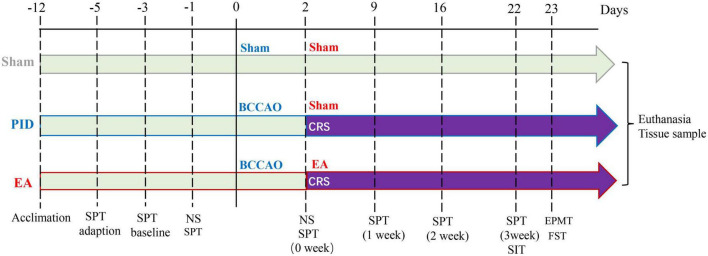
Schematic illustration of the experimental timeline and design. Male C57BL/6J mice were randomly assigned to three groups after 1 week of acclimation. Mice in each group have SPT adaption, SPT baseline measurement, and a preference test before the operation. The sham group was treated with a sham operation and sham EA (NA) treatment. The PID group was treated with BCCAO + CRS and sham EA (NA) treatment. The EA group was treated with BCCAO + CRS and EA treatment. The NS was applied 1 day before and 2 days after the operation to assess neurological deficits. Behavioral measures, including the SPT, SIT, EPM test, and FST, were conducted 21 days after CRS. SPT was recorded weekly for the entire experiment. After the behavioral tests, brain tissues were collected for further research. BCCAO, bilateral common carotid artery occlusion; CRS, chronic restraint stress; NS, neurological scoring; SPT, sucrose preference test; SIT, social interaction test; EPMT, elevated plus maze test; FST, forced swim test.

Cohort 1: A total of 41 mice were included in the study. However, five mice were excluded from further assessments due to death during or after surgery, and four mice were excluded because of the failure of ischemia induction. Additionally, one mouse (CD1) was used as a social target. Data were reported on 31 animals. After a seven-day adaptation period, the mice were randomly divided into three groups as follows. Sham group–2 days after the sham surgery procedure, the animals received non-acupoint (NA) toothpick puncture for 3 weeks (*n* = 12). PID group–2 days after BCCAO surgery, mice underwent restraint stress and received NA toothpick puncture for 3 weeks (*n* = 14); EA group–2 days after BCCAO surgery, mice underwent restraint stress and subsequently received EA treatment for 3 weeks (*n* = 14). The timeline of treatments, behavioral examinations, and tissue collection, is illustrated in Figure 1. Cohort 2: Twenty mice were included and underwent BCCAO surgery. However, three mice were excluded due to death during or after surgery, and two were excluded because of the failure of ischemia induction. The remaining 15 mice were randomly divided into three groups as follows: NA and saline-treated PID group (N/S-PID, *n* = 5), EA and saline-treated PID group (E/S-PID, *n* = 5), and Fluoxetine and NA-treated PID group (F/N-PID, *n* = 5). Two days after BCCAO surgery, mice in each group were subjected to restraint stress for 3 weeks. Group 1 received a daily gavage of 10 ml/kg of 0.9% saline and was treated with NA toothpick puncture for 3 weeks. Group 2 received a daily gavage of 10 ml/kg of 0.9% saline and was treated with EA stimuli for 3 weeks. Group 3 received a daily gavage of fluoxetine (2 mg/kg, Patheon, France) and underwent NA toothpick puncture for 3 weeks (Supplementary Figure 1). Investigators involved in the assessments were blinded to the group assignment during data collection and analysis.

### 2.3. Induction of transient global cerebral ischemia (GCI)

The transient bilateral common carotid artery occlusion (BCCAO) method was used to induce global cerebral ischemia in mice (Lu et al., 2020). Briefly, the mice were anesthetized with sodium pentobarbital (i.p., 50 mg/kg, Huaxia Chemical Reagent, China). Through a midline incision, the bilateral common carotid arteries were carefully exposed and separated from the carotid sheaths to prevent damage to the vagus nerves. To induce ischemia, BCCAs were temporarily occluded by micro-clips for 20 min. Afterward, the blood flow through the arteries was visually verified when the clips were removed. Rectal temperature was maintained at 36.5°C–37.5°C throughout the procedure with a heating blanket. Five minutes after reperfusion, the incisions were closed. Mice that exhibited dilated pupils, non-responsiveness to light during surgery, and a loss of the righting reflex for at least 30 s were selected for further experiments. Mice that did not meet these criteria were excluded. For the animals in the sham control group, identical procedures were performed, but the arteries were not occluded.

### 2.4. Neurological scoring

Neurological function was assessed using a scoring system detailed in Supplementary Table 1. A score of two is considered normal for each domain, with a maximum total score of 12. Assessments were performed 1 day before and/or 2 days after the BCCAO operation (Mai et al., 2019; Supplementary Figure 2).

### 2.5. Chronic restraint stress (CRS)

To induce restraint stress starting from the third day after BCCAO operation, mice in the corresponding groups were individually placed in 50 ml conical centrifuge tubes (3 cm in diameter and 10 cm in length) with holes at the bottom and cap for 1 h, twice per day (with a 2-h interval) during the light phase, for 21 consecutive days (Zhou X. et al., 2019). The control mice did not receive any stressful procedures but were handled daily in their home cages.

### 2.6. EA treatment

Electroacupuncture (EA) treatment started immediately after the first CRS exposure. Briefly, the mouse was fixed using hook and loop fasteners and restricted from movement with adhesive tape on its tail and neck. Then, the Baihui acupoint (GV 20, located at the intersection of the sagittal midline and the line linking the two ears) and the bilateral Zusanli acupoints (ST 36, located approximately 4 mm below the knee joint and 1–2 mm outside the anterior tibial tubercle) were stimulated by one-off sterile EA needles (0.18 mm × 13 mm, Huatuo, Shanghai, China) for 3 weeks (Monday through Friday each week) (Luo et al., 2017; Wang and Li, 2022). The needles were inserted 2–3.5 mm deep into acupoints. The stimulation was generated using an EA instrument (Model no. SDZ-III, Hwato, China), with an intensity of 0.5 mA and a frequency of 15 Hz for 30 min (Zhang et al., 2020). Mice were awake throughout the entire treatment. In the sham groups, toothpicks were attached to the NA (on the hips, located midway between the coccyx and hip joints) without skin penetration, and the animals did not receive any electrical stimulation.

### 2.7. Behavioral experiments

Animals underwent behavioral experiments, with less stressful tests preceding more stressful tests. The behavioral tests were performed in this order: SPT, SIT, EPM test, and FST.

#### 2.7.1. Sucrose preference test (SPT)

The procedure was performed according to a previously published protocol by Liu et al. (2018) with minor modifications. The SPT was divided into three stages: adaptation, baseline measurement, and preference test. During the adaptation stage, mice were kept in their home cages and given continuous access to two regular bottles containing sucrose water (1% wt/vol) for 48 h. During the baseline measurement stage, each mouse was given one tube of sucrose water (1% wt/vol) and one tube of regular water for a period of 12 h. The intake of sucrose and water was measured by weighing the two bottles. After a 12 h-interval, a second-round measurement was performed. After being deprived of both food and water for 16 h, mice entered the test stage. One bottle of 1% sucrose solution (100 ml) and one bottle of regular water (100 ml) were placed in each cage for 4 h, then the consumption rate of sucrose water was calculated. To avoid place preference, the positions of the two water bottles were alternated every 2 h. The tests were conducted and recorded before the BCCAO operation and at the end of each week after CRS exposure (Supplementary Figure 3). The reduction in sucrose consumption reflects increased anhedonia, and the results were analyzed using the following formula: sucrose preference (%) = sucrose solution consumption/total liquid intake × 100%.

#### 2.7.2. Social interaction test (SIT)

Social interaction test (SIT) consisted of two stages (Iniguez et al., 2014). In the first stage, each experimental mouse was allowed to freely explore a square open field arena (40 cm length × 40 cm width × 40 cm height) for 2.5 min, and the distance traveled (cm) during this session was recorded to examine the basal locomotor activity of the mice (Supplementary Figure 4). Along one side of the arena was a circular wire cage (7 cm diameter) that remained empty during this period. Afterward, the experimental C57BL/6J mouse was removed from the open field arena. In the second stage, an unfamiliar CD-1 mouse (target) was placed in the cage, then the experimental mouse was reintroduced, and the time (s) spent in the interaction zone (8 cm wide corridor surrounding the wire cage) was recorded. SI ratio = time spent in the interaction zone with a target/time spent in the interaction zone without a target. Reduced sociability was defined as SI < 1, while normal sociability was defined as SI > 1.

#### 2.7.3. Elevated plus maze (EPM) test

The EPMT apparatus was elevated to a height of 70 cm and consisted of two enclosed arms (50 cm × 10 cm × 6 cm) and two open arms (50 cm × 10 cm). At the beginning of each session, each experimental mouse was placed in the center of the maze, facing an open arm, and allowed to freely explore for 5 min. The time spent in closed and open arms was recorded. The anxiety level of animals was measured by calculating the average percentage of time they spent in the open arms (Xu et al., 2019).

#### 2.7.4. Forced swim test (FST)

Mice were forced to swim in a cylindrical transparent container (height 30 cm, diameter 15 cm) filled with water (height 15 cm, 23 ± 1°C). The duration of immobility time, whose increase is regarded as an indicator of despair and helplessness, was measured over the last 4 min of the 6-min test period. Each mouse was judged to be immobile when it remained floating passively in the water, slightly hunched but upright, with its head just above the surface. After the completion of each trial, the mouse was returned to the incubator, and the water was changed. FST was conducted only once for each animal at the end of the behavioral tests (Farajdokht et al., 2022).

### 2.8. Behavioral Z-score calculation

To obtain a comprehensive and integrated behavioral performance profile, we adopted the Z-score methodology (Guilloux et al., 2011). Z-scores are dimensionless mathematical tools that enable the normalization of results within studies and facilitate the comparison of related data across studies. Z-scores show how many standard deviations a given observation is above or below the mean of the control group, and the Z-score for each individual was calculated using the following formula:

Z=(X-μ)/σ

X represents the individual data for the observed parameter. μ and σ represent the mean and standard deviation of the control group, respectively. According to the formula, a higher positive Z-score indicates poorer performance. Z-scores were first computed from the behavioral data within each test and then aggregated across the tests to ensure equal weighting of the different tests. In this study, an overall behavior Z-score was obtained for each animal based on the four tests:

Behavior⁢score=[Z⁢(SPT)+Z⁢(SIT)+Z⁢(EPMT)+Z⁢(FST)]/4

### 2.9. Mouse euthanasia and tissue sample preparation

At the end of the procedure, the mice were deeply anesthetized with pentobarbital (50 mg/kg i.p.) and perfused intracardially with 30 ml of cold 0.9% normal saline, followed by 50 ml of cold 4% paraformaldehyde phosphate buffer (PFA, BOSTER, China). Intact brains were rapidly harvested and then post-fixed in 4% PFA at 4°C for 36 h and subsequently dehydrated in 20 and 30% sucrose for more than 24 h, respectively. After the brains had sunk to the bottom of the container, they were embedded and frozen on dry ice before being stored at −80°C. Sections were cut at a thickness of 30 μm in the coronal plane using a freezing microtome (Leica CM 1950 cryostat).

### 2.10. Immunohistochemistry and image acquisition

For immunofluorescent staining, brain slices containing the prefrontal cortex (PFC) region (anteroposteriorly 2.30 mm anterior to bregma), CA1 and CA3 regions (anteroposteriorly 1.76 mm posterior to bregma) of the hippocampus, and the parietal cortex (PC) region (anteroposteriorly 2.06 mm posterior to bregma) were washed with a 0.01 M PBS solution (PBS, BOSTER, China), then permeabilized with 0.3% Triton X-100 (Beyotime Biotechnology, China) for 15 min, and blocked with 10% goat serum albumin (BOSTER, China) for 1 h at room temperature. Sections were incubated with primary antibodies anti-GFAP mouse (1:500, Santa Cruz, USA) at 4°C overnight. After rewarming for 1 h at 37°C, the slices were washed three times (10 min each) with 0.01 M PBS. Subsequently, they were incubated with the secondary antibodies (goat anti-mouse Alexa Fluor 488, 1:200, Proteintech, China) for 2 h at room temperature (RT). Then, brain slices were incubated with DAPI (Beyotime Biotechnology, China) for 5 min in the dark at RT to stain the nuclei. Further, brain slices were mounted with an anti-fade mounting medium (Beyotime Biotechnology, China) and observed under a fluorescence microscope (VS200; Olympus, Japan) and a confocal microscope (A1R, Nikon, Japan). Three fields that displayed consistent and strong GFAP staining were selected from four regions of each mouse. GFAP-positive cells were then captured at 20 and 40× magnification. To ensure accurate appearance and measurements, the selected cells (*n* = 5 per mouse) must meet the following criteria: (a) they should be non-overlapping with neighboring cells, and (b) their branches must be visible. The *Z*-axis diameter for each cell was determined by identifying the upper and lower Z-planes, and the midpoint of the astrocyte was set as Z 0. The *Z*-axis step size was 1 μm.

### 2.11. Astrocyte morphology and Sholl analysis

All image processing was performed using the Fiji-ImageJ software (Schindelin et al., 2012). First, the raw files were imported, and the GFAP channel was separated by the Split tool. Z-stacks were projected into a two-dimensional image using the maximum intensity projection tool. The resulting images were then converted to an 8-bit format. After optimizing for brightness and contrast, the GFAP signal was segmented using the threshold tool. The resulting image was then processed with the despeckle tool, which effectively removed salt and pepper noise that could appear after thresholding (Codeluppi et al., 2021). Afterward, the cell images were converted to binary masks. Cell skeletonization was performed using the skeletonize tool, which allowed for the determination of process length and identification of any possible bifurcations. The number of branches [the processes surging from the soma of the cell or from a branch point (node), as shown in Figure 4E], endpoints (the terminals of final branches, as shown in Figure 4F), and the total length of branch processes (the combined length of all individual processes, as shown in Figure 4G) were measured using the Skeleton plugin. Convex hull perimeter (CHP, the perimeter that encloses the entire cell silhouette, as shown in Figure 4H) and convex hull area (CHA, the polygon made of straight lines that encloses the entire cell silhouette, as shown in Figure 4I) were measured with the FracLac plugin. The Sholl analysis plugin was used to create concentric circles (with fixed radius steps = 13) around the cell, starting from the soma and extending outward at increasing radial increments of 5 μm. The number of intersections, which are the points where the astrocytic processes cross concentric rings, was calculated. To calculate the total number of Sholl intersections, we summed up the intersections for each circle in an individual cell. All images were randomly coded and analyzed by three investigators who were blind to the experimental condition.

### 2.12. Statistical analysis

All statistical analyses were performed using GraphPad Prism 8 (San Diego, CA, USA). All data were expressed as means ± SD of n observations, where n represents the number of animals. Data with more than two groups were analyzed for statistical significance using either a one-way ANOVA test or a two-way ANOVA test, followed by Tukey’s test for multiple comparisons. Finally, the potential relationship between the complexity of cell morphology and behavioral performance (Z-scores) was determined using correlation analysis (Spearman r test). “Significance” was defined as having a *p*-value < 0.05.

## 3. Results

### 3.1. The assessment of behavioral changes

#### 3.1.1. The experimental condition of BCCAO and CRS induced anhedonia-like behavior in SPT, whereas EA prevented this effect

**FIGURE 2:**
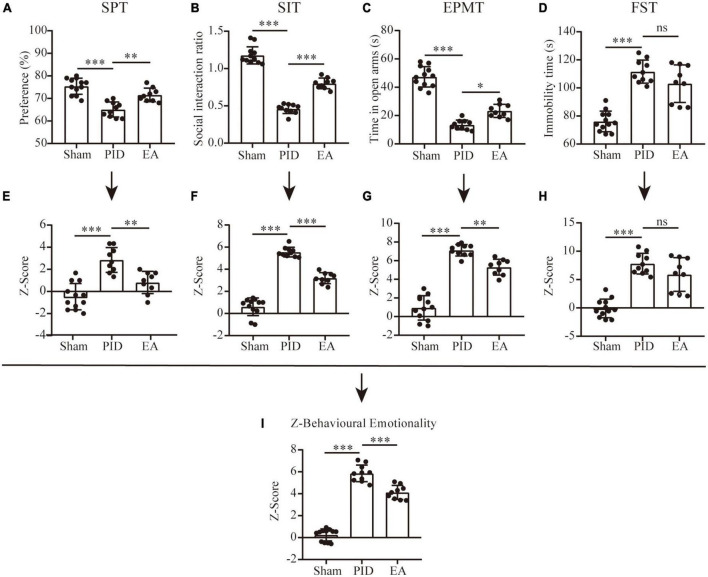
Behavioral performance and Z-scores. Dot-plot graphs show the individual values for the preference index of sucrose consumption **(A)**, social interaction ratio **(B)**, time spent in the open arms of the EPM **(C)**, and time spent immobile in the FST **(D)**. **(E–H)** Z-scores for the SPT **(E)**, SIT **(F)**, EPMT **(G)**, and FST **(H)**. **(I)** The overall Z-score of behavioral emotionality. All data are presented as mean ± SD. *n* = 9–12 mice per group. *p < 0.05, **p < 0.01, ***p < 0.001, and ns *p* > 0.05. This analysis was conducted using a one-way ANOVA.

In the SPT, no difference was observed in sucrose preference between groups in either the baseline measurement or the preference test before BCCAO surgery (Supplementary Figures 3A, B). However, after BCCAO surgery and 21 days of CRS exposure, mice in the PID group showed significantly decreased sucrose preference compared to the sham group, and this reduction of sucrose consumption was significantly improved by 3 weeks of EA treatment, measured in the last week of testing (PID: 65.10 ± 3.35 vs. 75.42 ± 3.58 in sham, *p* < 0.001; EA: 71.22 ± 3.46 vs. 65.10 ± 3.35 in PID, *p* < 0.01, Figure 2A). The weekly performances are shown in Supplementary Figure 3C.

#### 3.1.2. The beneficial effects of EA on sociability in the SIT following BCCAO and CRS

In the SIT, during the target-absent period, we found no differences in the total distance traveled between the groups (Supplementary Figure 4). As shown in Supplementary Figure 5, we recorded the time mice spent in social interaction zones before and after the targe present in. When the target was present, mice in the sham group showed significantly higher time spent in the social interaction zones and the SI ratio > 1 (target absent versus present: 80.50 ± 8.23 vs. 94.42 ± 11.69, *p* < 0.05). In contrast, the social interaction level was reduced in mice exposed to BCCAO and CRS, for the time spent in the social interaction zones was significantly lower with the presence of a social target and the SI ratio < 1 (target absent versus present, 72.2 ± 7.10 vs. 33.20 ± 6.48, *p* < 0.001). Significantly, mice in the EA group also manifested decreased social interaction, as they spent comparative time in the social interaction zone in the presence of the social target (target absent versus present, 73.11 ± 15.55 vs. 59.67 ± 14.39, *p* > 0.05). However, compared to the PID group, they have improved social interaction, which is revealed by a relatively higher SI ratio (PID: 0.46 ± 0.06 vs. 1.18 ± 0.12 in sham, *p* < 0.001; EA: 0.80 ± 0.07 vs. 0.46 ± 0.06 in PID, *p* < 0.001, Figure 2B).

#### 3.1.3. EA improved anxiety-like behavior in the EPM test in PID mice

As Figure 2C shows, when compared to sham mice, mice in the PID group presented an obvious anxiety-like behavior as revealed by a significantly decreased time spent in open arms in the EPM. Nevertheless, this reduction was markedly prevented by EA treatment, indicating EA was effective in ameliorating anxiolytic behavior induced by BCCAO and CRS (PID: 13.40 ± 3.41 vs. 47.33 ± 7.19 in sham, *p* < 0.001; EA: 23.33 ± 4.66 vs. 13.40 ± 3.41 in PID, *p* < 0.05).

#### 3.1.4. Mice treated with BCCAO and CRS displayed behavioral despair in the FST, but EA showed no significant effect on this alteration

The FST results revealed that total immobility time was increased considerably in the PID group when compared to the sham group, while EA treatment non-significantly altered the total immobility time of mice under PID state (PID: 111.6 ± 8.21 vs. 76.08 ± 7.34 in sham, *p* < 0.001; EA: 103.0 ± 13.37 vs. 111.6 ± 8.21 in PID, *p* > 0.05, Figure 2D). Since chronic stress-induced behavioral-despair in the FST is proven to be reversed by fluoxetine, an SSRI (Tang et al., 2022), we compared the effects of fluoxetine administration and EA on mice treated with BCCAO and CRS at the same time. As a result, fluoxetine administration that started at the onset of the CRS exposure significantly decreased the total immobility time of PID-exposed mice, but the EA group spent a similar immobile time compared with the non-treated group, suggesting EA may have no effect on behavioral despair induced by BCCAO and CRS (N/F-PID: 71.00 ± 9.54 vs. 103.8 ± 14.31 in N/S-PID, *p* < 0.01; E/S-PID: 92.80 ± 9.37 vs. 103.8 ± 14.31 in N/S-PID, *p* > 0.05. Supplementary Figure 6).

#### 3.1.5. Z-score analysis for behavioral changes

We employed Z-score analysis to investigate the potential of combining results across different behavioral tests of different groups based on the hypothesis that all the behavioral tests were similarly weighted. First, Z-score normalization was performed within the respective behavioral parameters, and the results showed there was a significant effect of BCCAO and CRS exposure on SPT, SIT, EPM test, and FST, as animals in the PID group displayed higher Z-scores than their controls (SPT, PID: 2.85 ± 1.12 vs. −0.47 ± 1.19 in sham, *p* < 0.001. SIT, PID: 5.55 ± 0.44 vs. 0.61 ± 0.80 in sham, *p* < 0.001. EPMT, PID: 7.11 ± 0.62 vs. 0.94 ± 1.31 in sham, *p* < 0.001. FST, PID: 7.80 ± 1.83 vs. −0.09 ± 1.63 in sham, *p* < 0.001). Then, except for the FST, there was a trend for significant effects of EA on SPT, SIT, and EPM test (SPT, EA: 0.82 ± 1.00 vs. 2.85 ± 1.12 in PID, *p* < 0.01. SIT, EA: 3.20 ± 0.50 vs. 5.55 ± 0.44 in PID, *p* < 0.001. EPMT, EA: 5.30 ± 0.85 vs. 7.11 ± 0.62 in PID, *p* < 0.01. FST, EA: 5.89 ± 2.97 vs. 7.80 ± 1.83 in PID, *p* > 0.05, Figures 2E–H). Finally, the average of normalized Z-score values generated a single value per mouse. As Figure 2I shows, the integrated analysis resulted in augmented statistical significances of the effects of BCCAO and CRS, as well as EA treatment (PID: 5.86 ± 0.76 vs. 0.25 ± 0.55 in sham, *p* < 0.001; EA: 4.12 ± 0.64 vs. 5.86 ± 0.76 in PID, *p* < 0.001).

### 3.2. The assessment of astrocyte morphological changes

#### 3.2.1. The effects of EA on the branching complexity of astrocytes in the PID state measured by classical Sholl analysis

**FIGURE 3:**
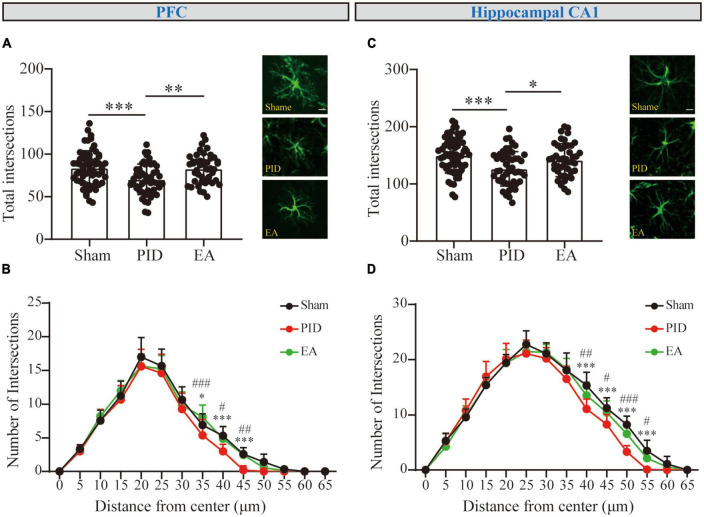
The branching complexity of astrocytes in the PFC and hippocampal CA1 regions. The mean number of total Sholl intersections of astrocytes and a representative GFAP-positive cell from each group in the PFC **(A)** and the hippocampal CA1 **(C)**. Those analyses were conducted using a one-way ANOVA. **(B,D)** Sholl distributive analyses of astrocytes in the PFC **(B)** and hippocampal CA1 region **(D)** in the sham group (black), PID group (red), and EA group (green). All data are presented as mean ± SD. *p < 0.05, **p < 0.01, and ***p < 0.001 compared to the sham group. #*p* < 0.05, ##*p* < 0.01, and ###*p* < 0.001 compared to the PID group. This analysis was conducted using a two-way ANOVA.

Glial fibrillary acidic protein (GFAP) constitutes the cytoskeleton of astrocytes and is commonly used as the marker staining the processes of astrocytes. We analyzed the morphological alterations of GFAP+ cells in brain areas including the hippocampus and the cortex, which are involved in mood regulation. To investigate the process complexity, the classical Sholl technique with fixed radius interval (5 um) and radii number (*n* = 13) was performed to provide information on the cell processes at a certain distance from the center of the cell soma. Under BCCAO and CRS exposure, astrocytes in the PFC and hippocampal CA1 regions displayed a decreased tendency in the total number of intersections among PID mice, compared to their sham mates (PFC, PID: 70.28 ± 17.89 vs. 83.57 ± 20.40 in sham, *p* < 0.001. CA1, PID: 126.3 ± 29.99 vs. in 149.6 ± 30.49 in sham, *p* < 0.001). After a 3-week period of EA treatment, PID mice exhibited a significant increase in the total number of intersections of astrocytes within the PFC and hippocampal CA1 regions, compared with mice in the PID group (PFC, EA: 82.82 ± 17.01 vs. 70.28 ± 17.89 in PID, *p* < 0.01. CA1, EA: 141.6 ± 28.86 vs. 126.3 ± 29.99 in PID, *p* < 0.05, Figures 3A, C). However, there was no significant effect of BCCAO and CRS exposure to astrocytes in the PC and hippocampal CA3 regions, and no significant difference was found after EA treatment in those regions either (Supplementary Figure 7).

#### 3.2.2. The effects of EA on distal intersections in PID state

Sholl curves were displayed to provide a direct appearance reflecting process distribution. The number of intersections for each concentric circle was calculated. For GFAP+ cells in the PFC (Figure 3B), the mean peak number of Sholl intersections was reached at approximately 20 μm distance from the soma for each group, and the mean number of intersections at the peak was not significantly different between the groups (*p* > 0.05). Nevertheless, we observed the mean number of intersections near the bottom was different between groups. Astrocytes in the PID group showed a significant reduction in the number of intersections at distal 35, 40, and 45 μm when compared with astrocytes in the sham mice, and this reduction was compromised by EA treatment, as astrocytes in EA-treated mice showed significantly increased intersections at distal circles (35 μm, PID: 5.40 ± 2.37 vs. 6.92 ± 1.68 in sham, *p* < 0.05; EA: 8.11 ± 1.76 vs. 5.40 ± 2.37 in PID, *p* < 0.001. 40 μm, PID: 3.00 ± 1.05 vs. 5.33 ± 1.37 in sham, *p* < 0.001; EA: 4.89 ± 1.76 vs. 3.00 ± 1.05 in PID, *p* < 0.05. 45 μm, PID: 0.20 ± 0.63 vs. 2.58 ± 1.00 in sham, *p* < 0.001; EA: 2.44 ± 1.13 vs. 0.20 ± 0.63 in PID, *p* < 0.01). In the hippocampal CA1 (Figure 3D), the mean peak number of Sholl intersections was reached at approximately 25 μm distance from the soma, and there was no significant difference in mean number of intersections at the peak between the groups (*p* > 0.05). Similar to what we had found in the PFC, there was a significant difference for astrocytic intersections at distal 40, 45, 50, and 55 μm between the sham group and the PID group. Specifically, a significant increase in the number of intersections was found between the PID and EA groups at distal radii (40 μm, PID: 11.1 ± 1.79 vs. 15.33 ± 2.39 in sham, *p* < 0.001; EA: 13.56 ± 2.35 vs. 11.1 ± 1.79 in PID, *p* < 0.01. 45 μm, PID: 8.30 ± 1.77 vs. 11.25 ± 1.87 in sham, *p* < 0.001; EA: 10.44 ± 2.13 vs. 8.30 ± 1.77 in PID, *p* < 0.05. 50 μm, PID: 3.30 ± 1.16 vs. 8.25 ± 1.55 in sham, *p* < 0.001; EA: 6.56 ± 2.01 vs. 3.30 ± 1.16 in PID, *p* < 0.001. 55 μm, PID: 0.10 ± 0.32 vs. 3.50 ± 1.93 in sham, *p* < 0.001; EA: 2.11 ± 0.93 vs. 0.10 ± 0.32 in PID, *p* < 0.05).

#### 3.2.3. The effects of EA on structural morphology of astrocytic processes in PID state

**FIGURE 4:**
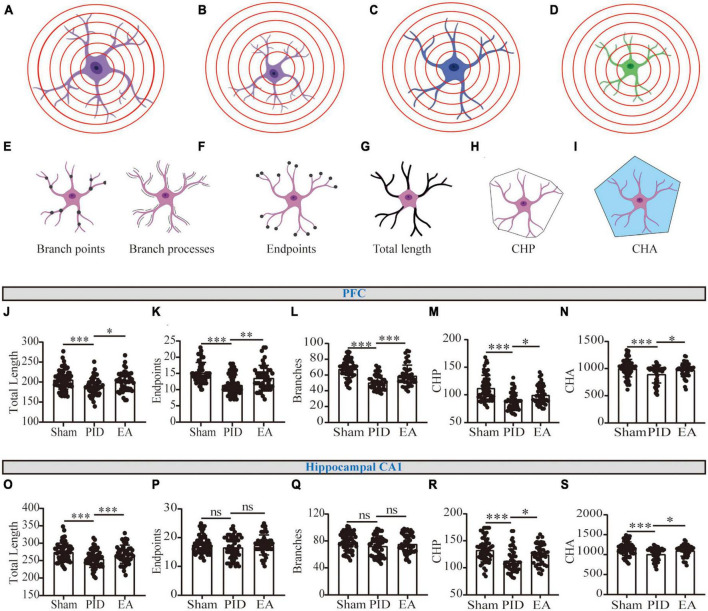
Astrocytic process morphology in the PFC and hippocampal CA1 regions. **(A)** Scenario illustration of an astrocyte. **(B–D)** Schematic representation of an astrocyte having similar branching complexity but smaller size **(B)**, less branching complexity but similar size **(C)**, or less branching complexity combined with smaller size **(D)**, compared to the astrocyte in **(A)**. **(E–I)** Schematic drawing of the astrocytic morphology for branch points and branch processes **(E)**, endpoints **(F)**, total length of processes **(G)**, CHP **(H)**, and CHA **(I)** per cell. **(J,O)** The total length of processes of astrocytes in the PFC **(J)** and the hippocampal CA1 region **(O)**. **(K,P)** The number of endpoints **(K)** of astrocytes in the PFC **(K)** and the hippocampal CA1 region **(P)**. **(L,Q)** The number of branch processes of astrocytes in the PFC **(L)** and the hippocampal CA1 region **(Q)**. **(M,R)** CHP of astrocytes in the PFC **(M)** and the hippocampal CA1 region **(R)**. **(N,S)** CHA of astrocytes in the PFC **(N)** and the hippocampal CA1 region **(S)**. Data presented as mean ± SD. *p < 0.05, **p < 0.01, ***p < 0.001, and ns *p* > 0.05. This analysis was conducted using a one-way ANOVA.

Theoretically, two main typical conditions in the branching processes may contribute to the shrinking of Sholl intersections (regardless of cell soma). For example, as to the astrocyte in Figure 4A, one condition is cell atrophy (Figure 4B), which represents cells that do not undergo the evident reduction in ramification, that is, no significant changes branch processes (Figure 4E) and endpoints (Figure 4F), but a decline in the total length of processes (Figure 4G). The other condition is the decline in ramification, which represents structural degradation (Figure 4C), and the comprehensive reductions in three main parameters including branch processes, endpoints, and total length of processes can indicate this manifestation. Of note, there exists combinative condition(s) (Figure 4D), that is, cells undergo structural simplification and atrophy, concurrently.

As a result, we observed that the GFAP+ cells in the PFC and hippocampal CA1 underwent a reduction in the total length of processes in mice exposed to BCCAO and CRS, compared with astrocytes in the control ones. Nevertheless, EA significantly improved the effect, as increased length of processes in the two regions was detected in EA-treated groups, compared to mice with no treatment in the PID groups (PFC, PID: 187.5 ± 21.17 vs. 206.5 ± 24.31 in sham, *p* < 0.001; EA: 200.6 ± 24.01 vs. 187.5 ± 21.17 in PID, *p* < 0.05. CA1, PID: 255.2 ± 24.93 vs. 275.1 ± 25.13 in sham, *p* < 0.001; EA: 268.5 ± 26.11 vs. 255.2 ± 24.93 in PID, *p* < 0.001, Figures 4J, O). In addition, by detecting endpoints and branches, we found PID mice displayed a significant reduction of branch arborization in the PFC, as shown by a significant decrease in the number of endpoints and branch processes. In contrast, after EA treatment, the consequences were significantly improved (Endpoints: PID: 11.50 ± 3.02 vs. 15.23 ± 3.16 in sham, *p* < 0.001; EA: 13.67 ± 3.82 vs. 11.50 ± 3.02 in PID, *p* < 0.01. Branches: PID: 50.42 ± 9.09 vs. 67.88 ± 11.16 in sham, *p* < 0.001; EA: 59.24 ± 13.35 vs. 50.42 ± 9.09 in PID, *p* < 0.001, Figures 4K, L). In the hippocampal CA1, the results indicate that PID mice showed a trend of decreased numbers in endpoints and branch processes, but this difference did not reach statistical significance (*p* > 0.05). In comparison with the PID group, the EA group showed no significant changes in the endpoints and branch processes of astrocytes in this region (Figures 4P, Q). Furthermore, CHP and CHA were used for estimating the changes in cell size. Figures 4M, N, R, S demonstrate both in the PFC and hippocampal CA1; the astrocytes in PID mice had significantly smaller convex perimeters and convex areas when compared with the astrocytes of sham mice. In contrast, the EA-treated mice exhibited an increase in the two parameters evaluated for the astrocytes in the PFC and hippocampal CA1 regions, compared to NA-treated mice (PFC: CHP: PID: 88.32 ± 15.39 vs. 112.4 ± 22.96 in sham, *p* < 0.001; EA: 99.91 ± 17.10 vs. 88.32 ± 15.39 in PID, *p* < 0.05. CHA: PID: 898.1 ± 164.1 vs. 1,007 ± 160.5 in sham, *p* < 0.001; EA: 977.1 ± 123.2 vs. 898.1 ± 164.1 in PID, *p* < 0.05. CA1: CHP: PID: 112.9 ± 21.79 vs. 132.8 ± 21.91 in sham, *p* < 0.001; EA: 124.5 ± 18.84 vs. 112.9 ± 21.79 in PID, *p* < 0.05. CHA: PID: 1,013 ± 155.7 vs. 1,150 ± 171.6 in sham, *p* < 0.001; EA: 1,102 ± 140.9 vs. 1,013 ± 155.7 in PID, *p* < 0.05).

### 3.3. The correlation between morphometric changes and Z-behavioral scores

**FIGURE 5:**
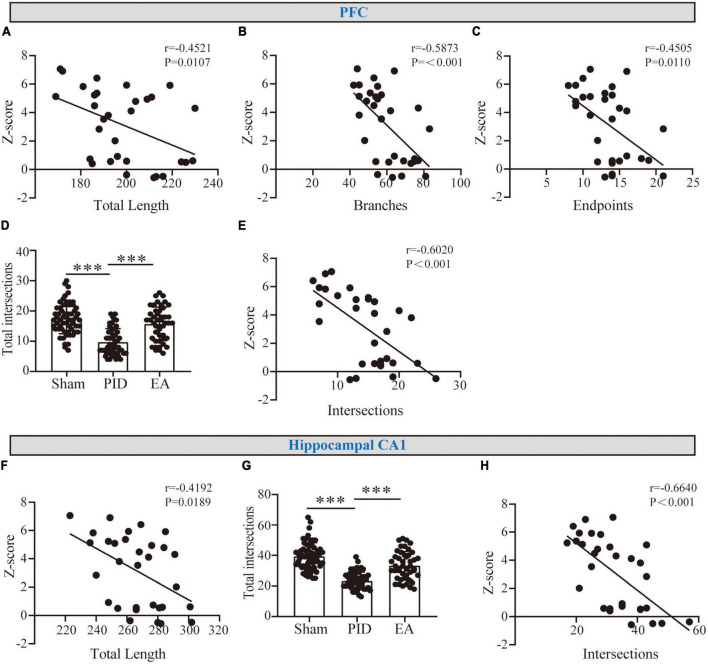
The correlation between astrocyte morphology and behavioral performances. **(A–C)** Graphical representation of the correlation between the total length of processes **(A)**, branches **(B)**, and endpoints **(C)** of astrocytes in the PFC with behavioral Z-scores. **(F)** Graphical representation of the correlation between the total length of processes of astrocytes in the hippocampal CA1 with behavioral Z-scores. r and *p*-values were obtained following a Pearson correlation analysis. **(D,G)** The sum of astrocyte intersections at distal radiuses from 35 to 65 μm in the PFC **(D)** and at distal radiuses from 40 to 65 μm in the hippocampus CA1 **(G)**. Data presented as mean ± SD. ***p < 0.001, one-way ANOVA. **(E,H)** The correlation between distal intersections of astrocytes in the PFC **(E)** and the hippocampal CA1 region **(H)** with behavioral Z-scores. r and *p*-values were obtained following a Pearson correlation analysis.

Based on the findings above, astrocyte processes showed evident atrophy in the hippocampal CA1, and atrophy was associated with deramification in the PFC after PID exposure, while EA effectively reversed those changes. To determine whether the observed alterations were associated with neurological function, the potential correlations between changed morphometric indicators and Z-behavioral emotionality scores were then investigated. As shown in Figures 5A–C, in the PFC, we found a strong and significant correlation for process length, branches, and endpoints with behavioral Z-score. In parallel, we found the astrocytic changes in process length in the hippocampal CA1 to be strongly and negatively correlated with behavioral Z-Scores (Figure 5F). Importantly, to further investigate whether the changed complexity in distal processes of astrocytes was related to behavioral performance, we measured the sum of astrocyte intersections at 35–45 μm in the PFC (Figure 5D), and at 40–55 μm in the hippocampal CA1 regions (Figure 5G) (PFC, PID: 9.78 ± 4.37 vs. 17.57 ± 5.09 in sham, *p* < 0.001, EA: 15.73 ± 5.55 vs. 9.78 ± 4.37 in PID, *p* < 0.001. CA1, PID: 23.36 ± 5.57 vs. 39.55 ± 8.74 in sham, *p* < 0.001; EA: 33.42 ± 9.17 vs. 23.36 ± 5.57 in PID, *p* < 0.001). As a result, we found that there is a strong and significant correlation between the number of distal intersections with the Z-scores of each mouse in both regions (Figures 5E, H).

## 4. Discussion

Our results determined that EA prevented depressive-like behavioral deficits and structural abnormalities in astrocytes in an experimental model of PID that combined BCCAO and CRS exposure. Furthermore, our findings showed a strong correlation between the behavioral Z-scores and the observed morphological changes and highlighted the primary role of astrocytic branch morphology in PID and EA-protected behaviors.

Global cerebral ischemia (GCI) is a complex disorder that results from a wide range of pathologic events, including asphyxia, arrhythmia, cardiac arrest, and shock (Neumann et al., 2013; Farajdokht et al., 2022). Due to the susceptibility of regions such as the hippocampus and cortex to ischemic insults, GCI is commonly accompanied by psychiatric disorders (Hu et al., 2023). However, from an etiological standpoint, the physical and psychological stress caused by movement restriction and high dependency on caregivers may lead to intense negative emotions in subjects (Farajdokht et al., 2022). Here, except for CRS paradigm may be superior to the chronic unpredictable mild stress (CUMS) exposure, which employs milder, randomized, and variable stressors to mimic the depressive state in most patients post-ischemia, we opted for this model as neurons had been shown to exhibit robust decreases in dendritic length and spine density after exposure to increased spatial restraint (Rico et al., 2015; Anderson et al., 2020; Codeluppi et al., 2021). As predicted, our data showed that mice treated with BCCAO and CRS exhibited depressive-like behaviors, including anhedonia (as measured by the SPT), behavioral despair (as measured by the FST), social reduction (as measured by the SIT), and anxiety (as measured by the EPM test).

Previous clinical and pre-clinical practices tended to use acupoint combinations for the treatment of stroke and/or depression (Ye et al., 2017; Mei et al., 2020; Lin et al., 2023). Based on the guidance of traditional Chinese medicine (TCM) theory and contemporary studies (Ye et al., 2017; Yang et al., 2022), we selected GV20 and ST36, not only for the two acupoints that are frequently used for the treatment of neurological and psychiatric diseases (Yang et al., 2022). Additionally, the combined stimulation of local (GV20) and distal (ST36) acupoints may have a synergetic effect on alleviating neuronal apoptosis, oxidative stress, and inflammatory responses (Torres-Rosas et al., 2014; Wang et al., 2014; Ye et al., 2017). All of these factors may also be crucial for the pathogenesis of ischemic insult, as well as the following PID (Wang et al., 2021; Farajdokht et al., 2022; Li et al., 2022). Consequently, we demonstrated that EA treatment had a beneficial effect on reward, anxiety, and sociability in PID mice. However, unexpectedly, the behavioral despair performance, measured as FST immobility time, did not change significantly although a tendency toward a decrease was noted. Moreover, our results showing that fluoxetine could ameliorate FST immobility in this PID model ruled out the experimental procedure glitches and suggest that EA and fluoxetine treatments may have different neurologic mechanisms for regulating behavioral performances as proposed. On the other hand, to minimize potential discrepancies and reduce intrinsic variability in single tests, we applied Z-score, a simple mathematical and integrative tool, to phenotype animals across related behavioral tests and to offer analytical opportunities afterward (Guilloux et al., 2011). As a result, we found that EA significantly reversed emotional abnormalities induced by BCCAO and CRS. This was manifested as lower emotionality Z-scores from all behavioral tests, including the FST. Indeed, a recent systematic review and meta-analysis by Li et al. (2022) summarized that Chinese herbal medicine (CHM), another important component of traditional Chinese medicine, showed significant improvement in depressive-like behaviors and neurological function of post-stroke depression animals by regulating neurotrophic factors, hormones, and corresponding signaling cascades in regulating neuroinflammatory responses and promoting hippocampal neuron regeneration. However, in contrast, only limited research has recently investigated the antidepressant effect of EA in laboratory animals with PID (Guilloux et al., 2011; Cai et al., 2019; Dong et al., 2021). Our data may provide a basis for future studies of acupoint selection and behavioral analysis in the PID condition.

Depressive disorder is linked to neuronal dysfunction in multiple brain regions (Pandya et al., 2012), while GCI often affects the forebrain of subjects (Wang et al., 2020). In the present study, we selected the hippocampal CA1 and CA3 regions as the neurons in the two sites are peculiarly prone to have neuroplastic impairments induced by chronic stress (Qiao et al., 2014; Liu et al., 2017). Besides, cortex areas including the PFC and PC were chosen for analysis due to their crucial role in regulating emotions through integrated functions such as decision-making, attention, and sensorimotor processing (Vichaya et al., 2019; Diaz-Castro et al., 2021). Considering that neurons and astrocytes have wide spatial and functional interactions, astrocytes in those areas may be more susceptible to undergo disturbance. However, our observations showed that BCCAO and CRS did not have a significant effect on the number of astrocytes in those areas (data not shown), and this finding contradicts previous literature that suggests a decrease in astrocyte cell density in MDD (Ongur et al., 1998; Cotter et al., 2002; Gittins and Harrison, 2011) despite sporadic research putting forward controversial conclusions supporting the findings that astrocyte cell density may not be affected by stress (Gittins and Harrison, 2011; Tynan et al., 2013). Another possible explanation for this phenomenon is that there, perhaps, exists some compensational or neutralizing factors, for example, neuroinflammation-induced astrogliosis in the early-stage but decreased GFAP protein synthesis or cell apoptosis, resulting in lower cell density in the late-stage (Verkhratsky et al., 2014), which may obscure the changes in cell number. Nevertheless, more research in this field is required in the future. Moreover, our evidence did not find a significant difference in the branching complexity of astrocytes in the PC and hippocampal CA3 regions compared to their respective controls. In contrast, astrocytes in the PFC and hippocampal CA1 presented morphological changes in response to BCCAO and CRS exposure, and EA effectively reversed these alterations. It is known that astrocyte populations are relatively homogeneous within one site but heterogeneous between different brain regions (Zhou B. et al., 2019). We infer that the astrocytic processes in the PFC and hippocampal CA1 region may be more susceptible or sensitive to ischemic insult when combined with chronic stress, compared to those in the PC and hippocampal CA3 region. Alternatively, the latter two regions may be relatively resilient to the stimuli in our experimental paradigm. Otherwise, the structural remodeling of astrocytes in different regions may be triggered asynchronously by genetic or other factors, which may also contribute to this discrepancy (Salois and Smith, 2016; Park et al., 2018; Sarkar and Biswas, 2021). At the very least, our results suggest that astrocyte morphological abnormality is not a diffuse phenomenon in the PID condition.

The Sholl technique is a credible method to quantify dendritic morphology (Gomez et al., 2018; Althammer et al., 2020; Codeluppi et al., 2021). By measuring the number of intersections, cell arborization, and complexity can be well evaluated, and the abnormality in this indicator has been proven to be related to numerous cognitive or stressful diseases (Zappa Villar et al., 2018; Codeluppi et al., 2021; Lin et al., 2023). In this study, we adapted classical Sholl analysis to quantify intersections with a fixed number of radii and fixed intervals. In theory, the decrease in the total number of intersections can be attributed to two main causes: cell atrophy and reductions in process branching (Zhang et al., 2015; Zappa Villar et al., 2018). For GFAP+ cells in the hippocampal CA1 regions, there was a decrease in the number of intersections and the total length of processes but no significant change in the number of branching processes and endpoints, suggesting that astrocytes may undergo process atrophy in this region. By the analysis of CHP and CHA, we conclude that astrocytes may undergo whole-cell atrophy. For GFAP+ cells in the PFC, the overall reduction in all parameters indicates that astrocytes undergo both atrophy and apparent process de-ramification or reorganization following BCCAO and CRS. Importantly, astrocytes often experience morphological alteration in their processes in a manner from far to near (Tynan et al., 2013; Codeluppi et al., 2021), so this may not be surprising that intersections in distal processes show significant differences between groups. Furthermore, tiny processes like peri-synaptic astrocyte processes (PAP), are barely detected by GFAP immunolabeling (Zhou B. et al., 2019; Codeluppi et al., 2021). Our data were more likely to provide a possible underestimation of the degree of real cell atrophy and structural remodeling rather than an overestimation. In most cases, the reduction in astrocytic complexity caused by repeated exposure to stress or other chronic pathological stimuli occurs gradually and persistently (Zappa Villar et al., 2018; Althammer et al., 2020), thus exposing PID mice to EA treatment for a period of 3 weeks, overlapping with the progression of stressful insult, which may reverse some of the morphological pathology, but the exact mechanisms involved remain unclear based on our present study. According to previous studies, molecular mechanisms including increased extra-synaptic glutamate and metabotropic glutamate receptors (mGluRs), high glucocorticoid (GC) levels, decreased trophic support, and the opening of gap-junction channels may drive the changes in astrocytic process motility and a decrease in structural plasticity (Popoli et al., 2011; Gomez et al., 2018; Zhou B. et al., 2019). Further studies to understand the influence of EA on astrocyte morphology can also be conducted in those directions. Here, we collected clues from previous studies that support the involvement of some of the aforementioned molecules and their related pathways in the antidepressant effect of EA in other models of depression. For instance, You et al. (2010) reported that EA effectively down-regulated the GC content and GC receptor mRNA expression in the hippocampus in a chronic depression model; EA relieved depressive-like behaviors after middle cerebral artery occlusion (MCAO), which was related to its activation in the expression of brain-derived neurotrophic factor (BDNF) and its receptor TrkB in the hippocampus (Kang et al., 2021); by regulating the glutamate and NMDA receptors, EA preserved neurons in the hippocampus and prevented CUMS-induced depressive behavior (Guo et al., 2021). Furthermore, EA may play a role in depression or mood disorders by stimulating the subcortical nuclei through the activation of noradrenergic or dopaminergic systems, which are known to stimulate specific astrocytic receptors to induce various signaling cascades (Hui et al., 2000; Lin et al., 2023). A recent observation demonstrating that EA restored expression of astrocyte-specific glutamate transporter EAAT2 which performs 90% of glutamate neurotransmission following chronic stress may decipher a mechanism translating acupuncture into changes in astrocytes that protect against depressive changes instigated by chronic stress (Luo et al., 2017). Nonetheless, there is no doubt that our knowledge of EA is still in its infancy. More detailed evidence is required to confirm whether these mechanisms account for EA in modulating astrocyte morphology.

Astrocytes are exquisitely sensitive to changes in their extracellular environment, and like neurons and microglia, their structure is intimately linked with their function (Gomez et al., 2018; Zhou B. et al., 2019; Althammer et al., 2020). The role of astrocyte morphology has been explored in cognitive and emotional diseases such as Alzheimer’s disease (AD), Parkinson’s disease (PD), and MDD (Villalba and Smith, 2011; Yeh et al., 2011; Rajkowska and Stockmeier, 2013), but seldom attention has been paid to the field of PID. To investigate the potential relationship between behavioral assays and astrocyte morphology in PID, we examined whether the altered morphological indicators that differed significantly among groups in the PFC and hippocampal CA1 regions were correlated with behavioral Z-scores. The significant correlation between the variables suggests that depressive-like behaviors may be influenced by the atrophy or deramification of astrocytic processes. Alternatively, astrocyte morphology could also be a result of neuronal dysfunction, which is the direct cause of behavioral deficits. However, specific manipulation of astrocytes and astrocytic homeostatic cascades can induce depression-like behaviors, which may help to support the former conclusion. Previously, Banasr and Duman (2008) utilized astrocyte-specific toxin L-alpha-aminoadipic acid and found that the ablation of astrocytes in the rat PFC induced depressive-like behavior similar to that observed following exposure to chronic stress; current research by Tynan et al. (2013) emphasized more elaborate changes in the structural plasticity of astrocytes in the PFC, for example, a ∼40% reduction in process length, or/and a ∼57% reduction in the branches, were sufficient to lead to behavioral abnormalities. Among the multiple morphological parameters, the number of distal intersections is extremely meaningful to indicate astrocyte function in synaptic regulation (Codeluppi et al., 2021). Although astrocytes cannot produce action potentials like neurons, the fine structure of astrocytes is found to be highly plastic and the distal protrusions comprise an essential part of the synaptic structure (Zhou X. et al., 2019). Regarding the huge quantity proportion in the CNS, the shortage or atrophy of astrocytic processes may lead to the disruption of the astrocyte network, which then extensively affects the connections with synapses and damages the neuronal function, thereby inducing mood disturbances and depressive behavior, ultimately (Tynan et al., 2013; Zhang et al., 2015; Codeluppi et al., 2021; Lin et al., 2023). This flow associated with process morphology may be one of the most important contributors of astrocyte dysfunction to many neurological and depressive disorders.

To our limited knowledge, we provided evidence for the first time that EA can be a potential candidate for modulating astrocyte morphology in PID. This finding is meaningful given the crucial role of astrocytic structure in maintaining normal emotional state and behavior. However, this study is not without limitations. First of all, our experiment only investigated male mice. Biological mechanisms underlying the development of ischemic insult and depression may vary by sex (Koga et al., 2020). To fully explore the effects of EA, it would be informative to conduct investigations using female mice. Second, since the task of morphological analysis is extremely time-consuming, we only focused on the main but limited parameters in our present study, which may lead to an incomplete description of the delicate structure of cells to some extent. We recommend new automatic morphology analysis software and more staining markers to be used for further analysis. Last but not least, we did not directly study neurons and synapses in this research; additional elaborate studies on the mechanisms are required to further elucidate the influence of EA in PID.

## 5. Conclusion

Our data demonstrating that the structural reorganization and/or atrophy of astrocytes was paralleled with behavioral performance in the PID experimental model, while EA alleviated behavioral deficits and restored those morphological alterations, provides new evidence to support the idea that EA protects PID from an astrocytic perspective.

## Data availability statement

The raw data supporting the conclusions of this article will be made available by the authors, without undue reservation.

## Ethics statement

The animal study was approved by the Ethics Committee of Chongqing Medical University. The study was conducted in accordance with the local legislation and institutional requirements.

## Author contributions

JW and XD: conceptualization, methodology, and software. JW and ZY: data curation. YJ and JW: formal analysis. YL and JJ: funding acquisition and resources. YL: project administration and supervision. JW: writing—original draft. XD, JJ, YJ, and YL: writing—review and editing. All authors have read and agreed to the published version of the manuscript.
