# Supplementary material for: Evaluation of electroacupuncture as a non-pharmacological therapy for astrocytic structural aberrations and behavioral deficits in a post-ischemic depression model in mice
Source: Front Behav Neurosci. 2023 Aug 28;17:1239024. doi: 10.3389/fnbeh.2023.1239024 (PMC10493307; doi:10.3389/fnbeh.2023.1239024)
Supplement: Supplementary file 1 [file Data_Sheet_1.pdf]

Supplementary Table.1

Supplementary Figure S1

Schematic illustration of the experimental timeline of the additional cohort. Male C57BL/6J mice were randomly assigned to three groups after 1 week of acclimation and BCCAO operation. NS was applied 2 days following BCCAO operation to assess neurological deficits. FST was performed after 21 days of CRS. NS, neurological scoring; FST, forced swim test.

Supplementary Figure S2

Neurological scores of mice. (A) no difference in NS between groups in cohort one at 1 day before operation. n=12-14 per group. (B) no difference in NS between PID group and EA group at 2 days after BCCAO operation. n=9-12 per group. (C) no difference in NS between groups in cohort two at 2 days after BCCAO operation. n=5 per group. Data presented as mean ± SD. ***p <0.001, ns p >0.05. This analysis was conducted using a one-way ANOVA.

Supplementary Figure S3

SPT baseline and the tests before operation or recorded weekly. (A) no difference in the ratio of sucrose consumption between groups measured in baseline-measurement period. (B) no difference in the preference test between groups before operation. (C) the performance of SPT measured weekly. n=9-14 per group. Data presented as mean ± SD. **p <0.01, ***p <0.001 compared with sham group. ##p <0.01, ###p <0.001 compared with PID group. ns p > 0.05. This analysis was conducted using a two-way ANOVA.

Supplementary Figure S4

The basal locomotor activity of mice in the SIT. No difference in locomotor activity was observed between the groups. n=9-12. Data presented as mean ± SD. ns p > 0.05. This analysis was conducted using a one-way ANOVA.

Supplementary Figure S5

Interaction time of mice in the SIT. Mice in the sham group spent significantly higher time in the presence of a social target, whereas mice in the PID group and EA group did not spend higher time in the target-in period. n=9-12. Data presented as mean ± SD. *p <0.01, ***p <0.001. ns p > 0.05. This analysis was conducted using a two-way ANOVA.

Supplementary Figure S6

FST immobile time of mice in the cohort two. n=5. Data presented as mean ± SD. **p <0.01, ns p >0.05. This analysis was conducted using a one-way ANOVA.

Supplementary Figure S7

The branching complexity of astrocytes in the PC and hippocampal CA3 regions. Data presented as mean ± SD. ns $p > 0.05$. This analysis was conducted using a one-way ANOVA.
