# Supplementary material for: Evaluation of electroacupuncture as a non-pharmacological therapy for astrocytic structural aberrations and behavioral deficits in a post-ischemic depression model in mice
Source: Front Behav Neurosci. 2023 Aug 28;17:1239024. doi: 10.3389/fnbeh.2023.1239024 (PMC10493307; doi:10.3389/fnbeh.2023.1239024)
Supplement: Supplementary file 2 [file Table_1.DOCX]

**Table S1. Neurological Function Score criteria.**

| Level of consciousness |  |
| --- | --- |
| 1. No reaction to pinching of tail | 0 |
| 1. Poor response to tail pinch | 1 |
| 1. Normal response to tail pinch | 2 |
| Corneal reflex |  |
| 1. No blinking | 0 |
| 1. Sluggish blinking | 1 |
| 1. Normal blinking | 2 |
| Respirations |  |
| 1. Irregular breathing pattern | 0 |
| 1. Decreased breathing frequency, normal pattern | 1 |
| 1. Normal breathing frequency and pattern | 2 |
| Righting reflex |  |
| 1. No turning attempts | 0 |
| 1. Sluggish turning | 1 |
| 1. Turns over spontaneously and quickly | 2 |
| Coordination |  |
| 1. No movement | 0 |
| 1. Moderate ataxia | 1 |
| 1. Normal coordination | 2 |
| Movement/activity |  |
| 1. No spontaneous movement | 0 |
| 1. Sluggish movement | 1 |
| 1. Normal movement | 2 |
| Total possible score | 12 |
