# Supplementary figures and images for: Evaluation of electroacupuncture as a non-pharmacological therapy for astrocytic structural aberrations and behavioral deficits in a post-ischemic depression model in mice
Source: Front Behav Neurosci. 2023 Aug 28;17:1239024. doi: 10.3389/fnbeh.2023.1239024 (PMC10493307; doi:10.3389/fnbeh.2023.1239024)

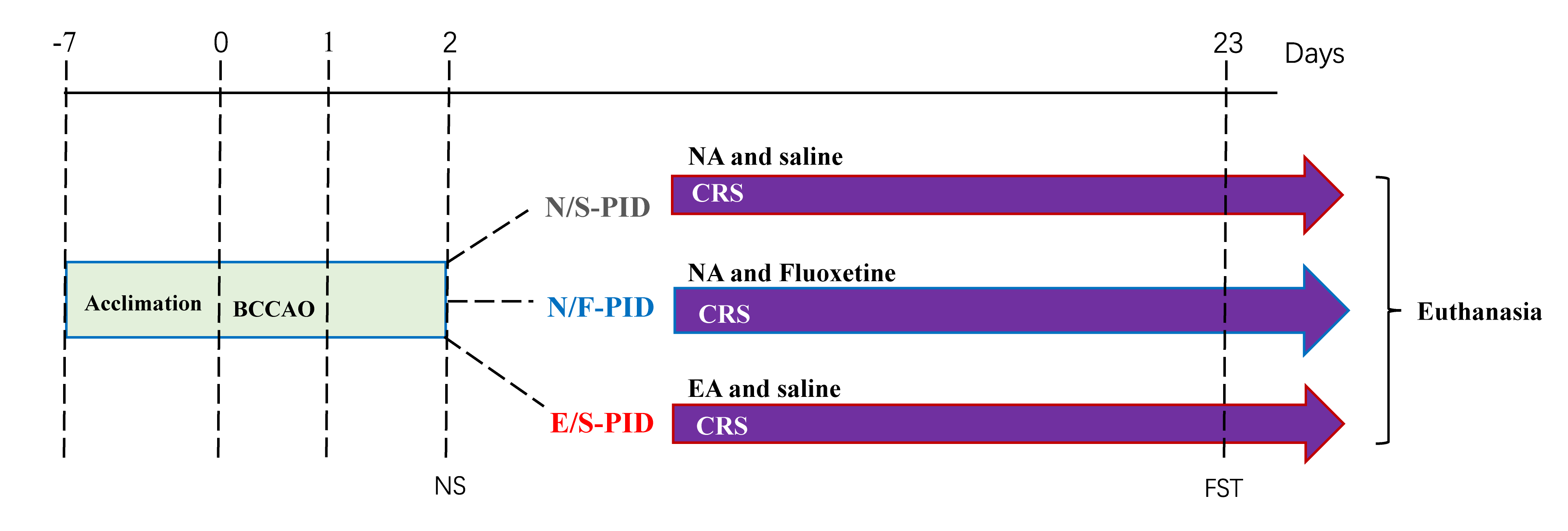

Supplement: Supplementary file 3 [file Image_1.TIF]

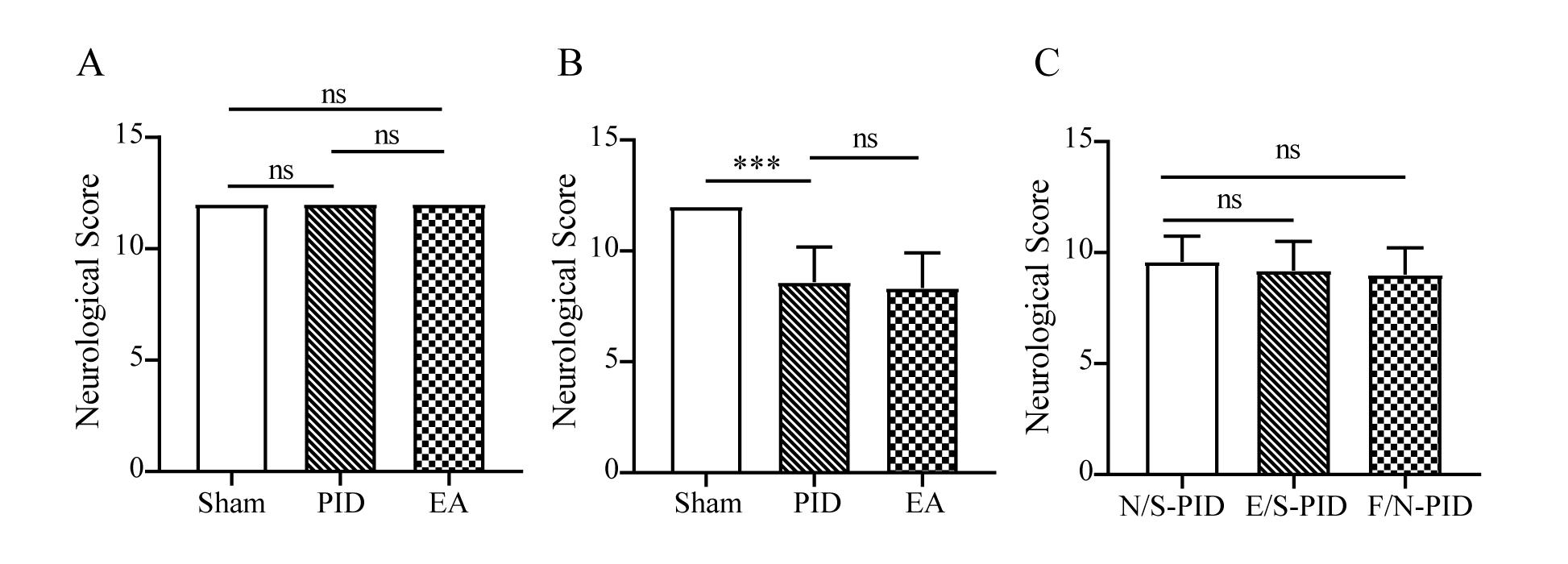

Supplement: Supplementary file 4 [file Image_2.TIF]

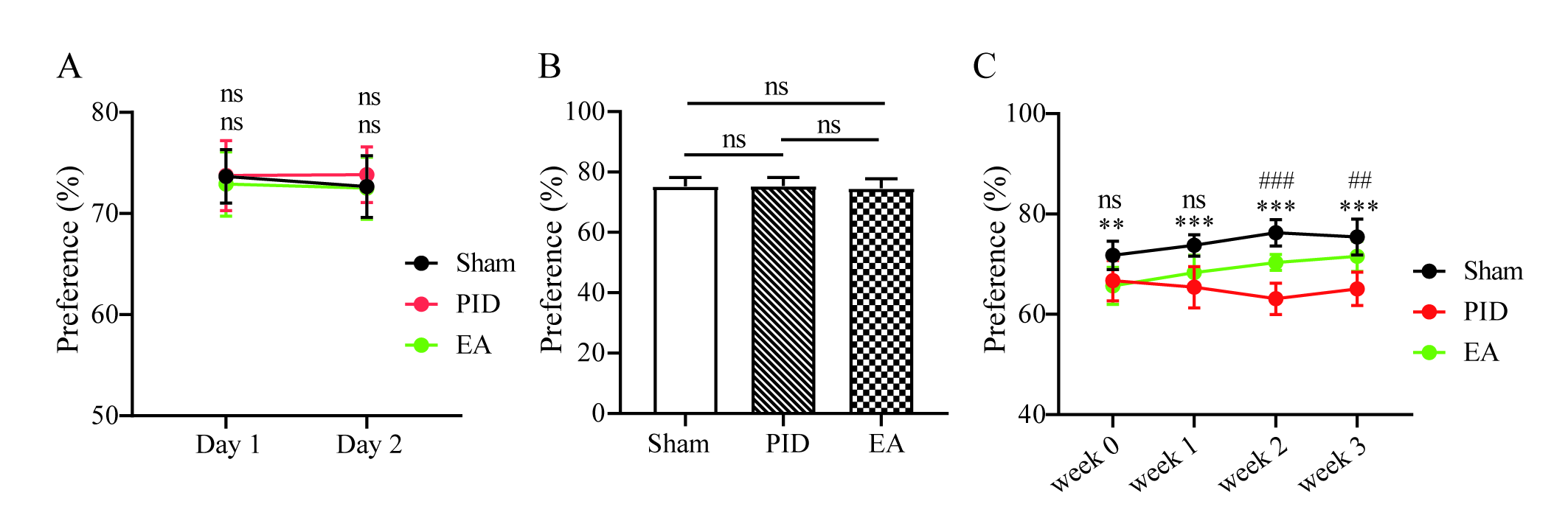

Supplement: Supplementary file 5 [file Image_3.TIF]

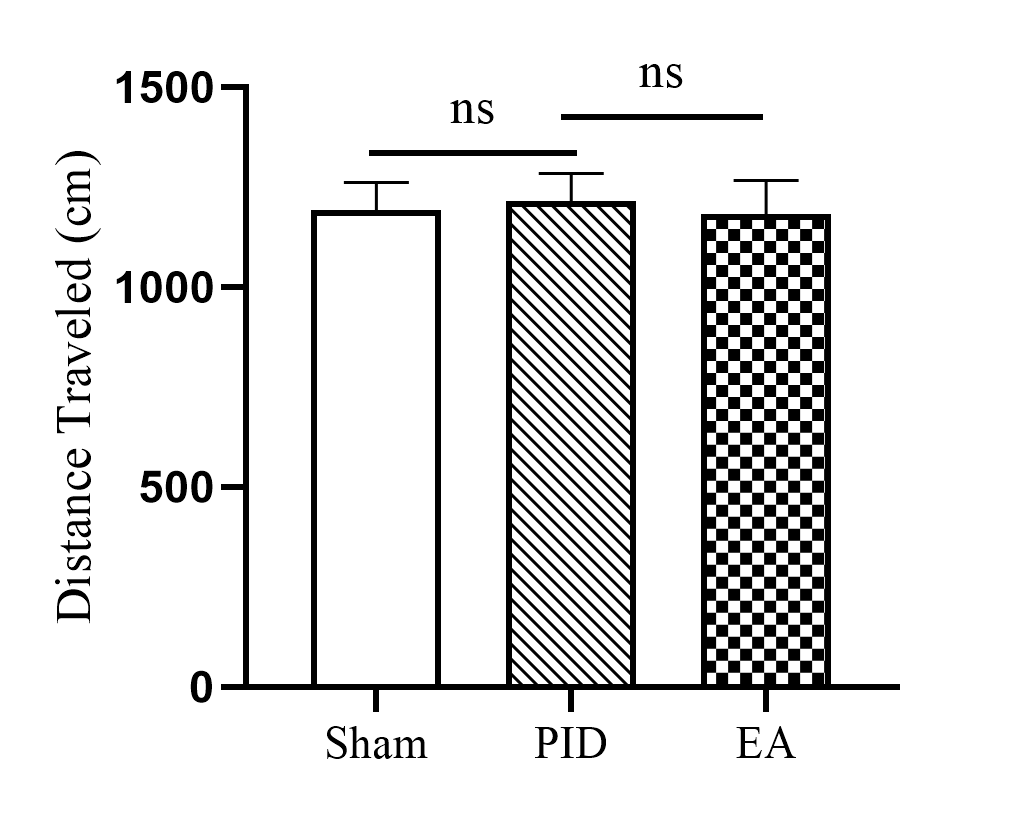

Supplement: Supplementary file 6 [file Image_4.TIF]

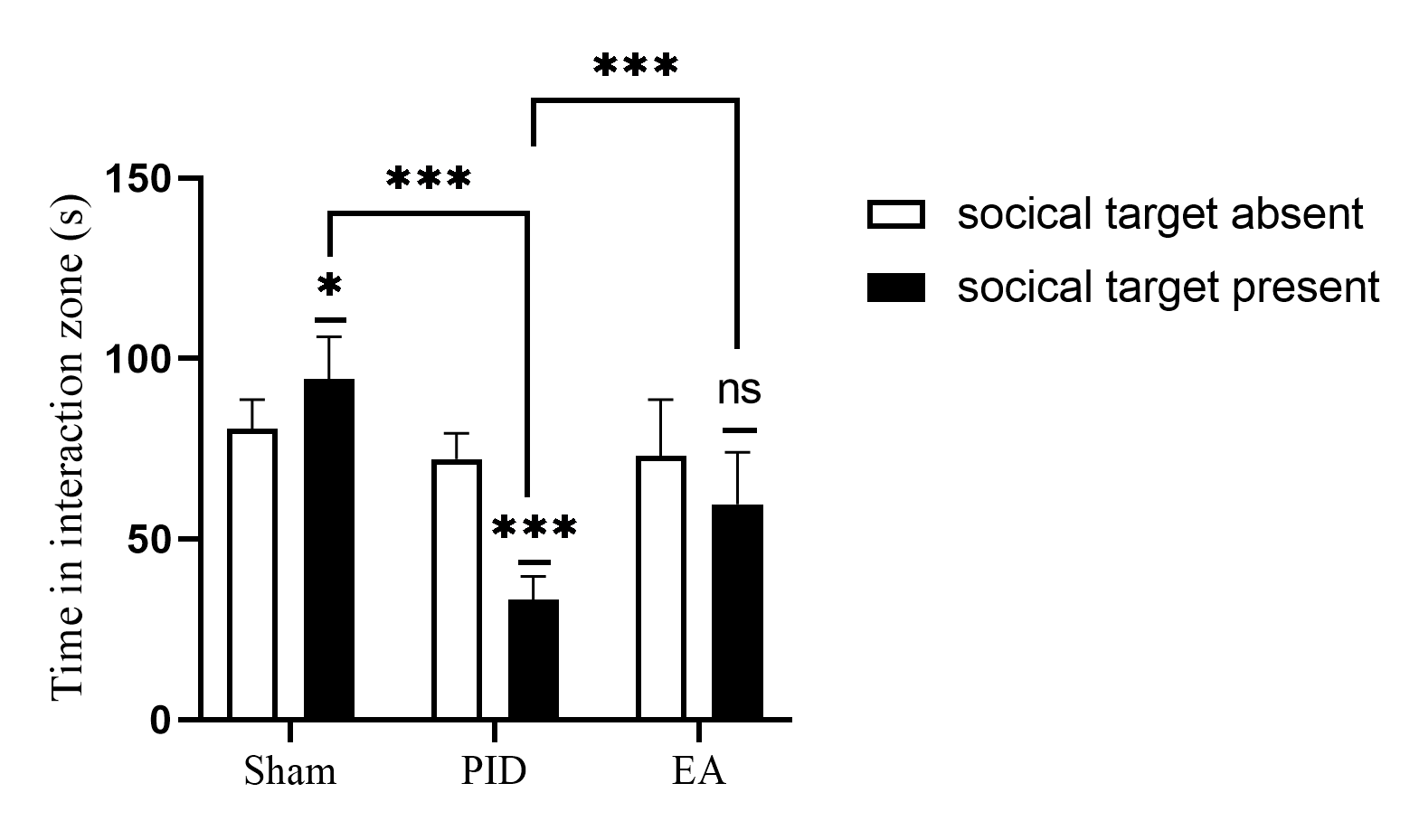

Supplement: Supplementary file 7 [file Image_5.TIF]

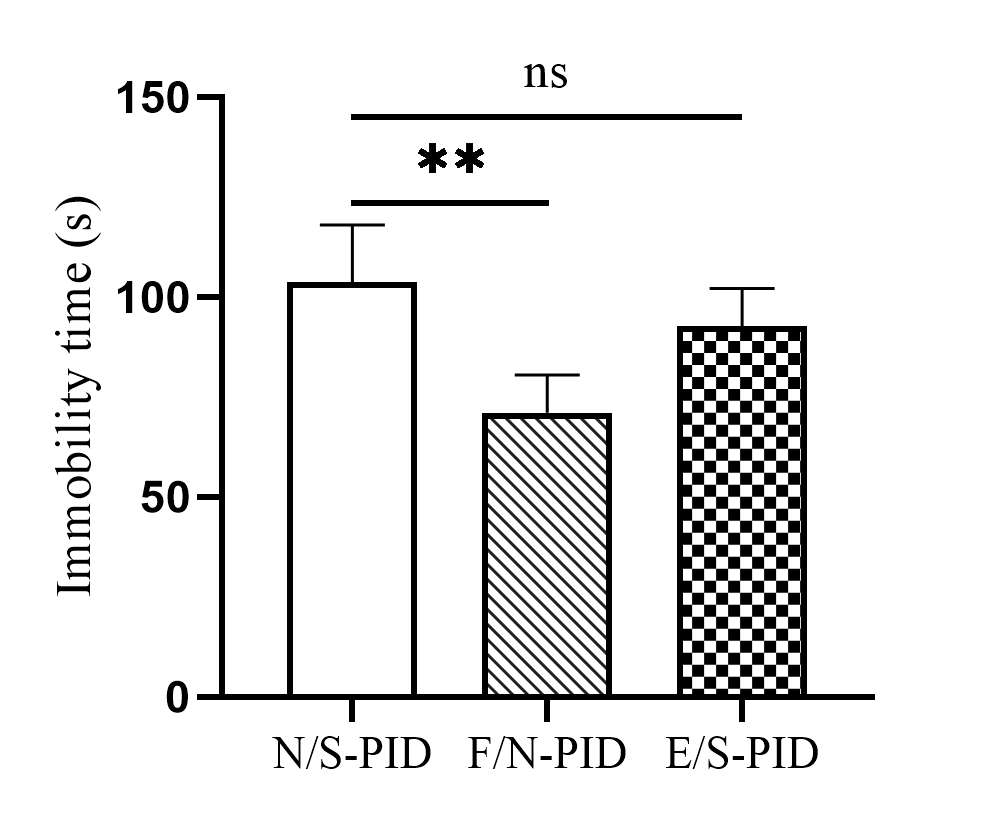

Supplement: Supplementary file 8 [file Image_6.TIF]

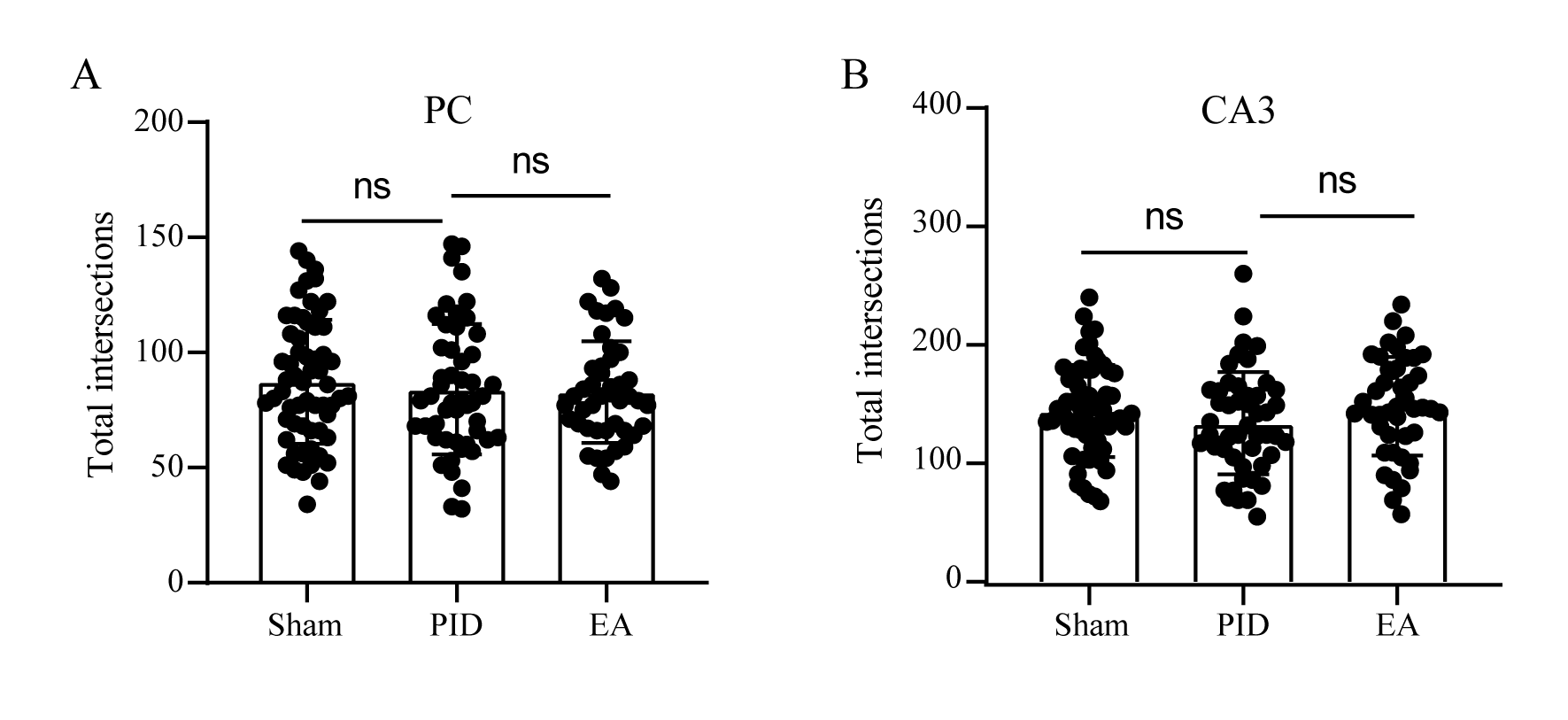

Supplement: Supplementary file 9 [file Image_7.TIF]
